# How do self-exempt beliefs affect intentions to quit smoking? An exploration of the mediating role of threat appraisal and coping appraisal

**DOI:** 10.3389/fpsyg.2023.1260561

**Published:** 2023-11-30

**Authors:** Dan Zhang, Runhua Liu, Xinchen Li, Yuanyuan Yuan, Geyao Zhou

**Affiliations:** ^1^School of Medicine and Health Management, Guizhou Medical University, Guiyang, China; ^2^Center of Medicine Economics and Management Research, Guizhou Medical University, Guiyang, China; ^3^Guizhou Provincial Institute of Health Development, Guizhou Medical University, Guiyang, China; ^4^Department of Management Engineering, Guizhou Traffic Technician and Transportation College, Guiyang, China

**Keywords:** self-exempt beliefs, intention to quit smoking, protection motivation theory, threat appraisal, coping appraisal, partial least squares, mediating effect

## Abstract

**Introduction:**

Numerous smokers are cognizant of the detrimental effects associated with this habit yet exhibit a persistent reluctance to cease their tobacco consumption. Self-exempt beliefs serve as an obstacle to the cessation of this addictive behavior. This research explored the impact of self-exempt beliefs on the readiness to quit smoking based on the Protection Motivation Theory (PMT) model and the mediating roles of threat appraisal and coping appraisal.

**Methods:**

Self-exempt beliefs, PMT constructs, and the intention to quit smoking constituted the theoretical model. The questionnaires were collected from 488 Chinese adult male smokers based on snowball sampling. Exploratory Factor Analysis (EFA) was used to examine the underlying factor structure of the pre-designed self-exempt beliefs scale. The reliability, validity, path coefficients, and explanatory power of the model were calculated using Partial Least Squares Structural Equation Modeling (PLS-SEM).

**Results and discussion:**

The results showed that : (1) three common factors (skeptic beliefs, bulletproof beliefs, and “worth it” beliefs) with a total of 11 items were retained after EFA; (2) skeptic beliefs and “worth it” beliefs had a significantly negative effect on both threat appraisal and coping appraisal, while bulletproof beliefs did not; (3) bulletproof beliefs had a significantly positive direct impact on intention to quit, “worth it” beliefs had a significantly negative direct impact on intention, while skeptic beliefs had no significantly direct impact on intention; (4) threat appraisal and coping appraisal positively and significantly predicted cessation intention; and (5) threat appraisal and coping appraisal, as two main cognitive processes, acted as full mediations between skeptic beliefs and the intention to quit, as complementary partial mediations between “worth it” beliefs and the intention, and as non-mediation between bulletproof beliefs and the intention. Our findings suggest that efforts to undermine or “prevent” these self-exempt beliefs, particularly “worth it” and skeptic beliefs, may be an effective tactic for health communication interventions for quitting smoking.

## 1 Introduction

Tobacco usage is still one of the most pressing problems in public health today. As a major producer of tobacco, China has smoked around 40% of the world’s cigarettes, primarily by males (Chinese Center for Disease Control and Prevention [CDC], 2020). It is estimated that tobacco consumption causes more than one million fatalities annually in China (Chan et al., 2022). The proportion of adult deaths attributable to male smoking is rising (Chen et al., 2015). If present rates persist, tobacco-related fatalities in China will surpass 2 million by 2030 and 3 million by 2050 (Chen et al., 2015). What is even grimmer is that smoking is shown to considerably increase the odds of 22 causes of mortality and 56 particular illnesses affecting all major organ systems, leading to avoidable causes of mortality (Li et al., 2021; Chan et al., 2022). Smoking has also been proven to be related to psychological disorders such as anxiety and depression (Husaini et al., 2017; Mathew et al., 2017; Najafipour et al., 2021). Smokers who do not give up the habit will experience the onset of old-age diseases about 10 years sooner than non-smokers (Jha and Peto, 2014). Quitting smoking before developing a life-threatening disease is extremely advantageous (Pirie et al., 2013; Chen et al., 2015; Chan et al., 2022). Thus, it is essential to encourage current smokers to develop the intention to quit.

However, conventional intervention strategies, such as promoting the negative effects of smoking, have had little effect on increasing smokers’ desire to quit (Pei and Yang, 2022). Despite broad awareness of the dangers and harms of tobacco smoking, a shocking proportion of smokers continue to engage in this lethal habit (Fotuhi et al., 2013; Guillaumier et al., 2016). Festinger’s (1957) classic Cognitive Dissonance Theory is often used to explain this inconsistency. When individuals exhibit actions that are inconsistent with their personal beliefs, they experience a psychological state characterized by cognitive dissonance, leading them to seek ways to alleviate this discomfort. The process of dissonance reduction tends to favor the selection of the path that offers the least amount of resistance. When faced with difficulty in achieving a behavioral change, such as smoking cessation, individuals tend to undergo a transformation in their attitudes instead. Given the considerable difficulty associated with smoking cessation (Hughes et al., 2004), the theory posits that individuals who smoke are more inclined to modify their beliefs in order to justify their actions rather than making actual changes to their smoking behavior (Festinger and Carlsmith, 1959; Fotuhi et al., 2013). Those beliefs are referred to as dissonance-reducing beliefs (Fotuhi et al., 2013), self-exempt beliefs (Chapman et al., 1993), risk denial (Peretti-Watel et al., 2007a), justifications (Sidhu et al., 2022), or rationalizations (Kleinjan et al., 2009; Lee et al., 2009; Huang et al., 2019; Sidhu et al., 2022) in different literature.

These beliefs are believed to be related to smoking cessation intentions or behaviors. In longitudinal studies, most studies made use of the data from the International Tobacco Control (ITC) of different countries, thus choosing the same belief indicators, including functional beliefs and risk-minimizing beliefs (Yong and Borland, 2008). A controversial question is whether the beliefs negatively influenced smoking cessation behaviors or whether the failure to quit promoted rationalizing beliefs. Some scholars examined the relationship between rationalization beliefs during one wave and cessation during the subsequent wave and concluded that two kinds of beliefs are negatively related to subsequent attempts to cease (Borland et al., 2009). Other scholars, however, examined the increase or decrease of pro-smoking beliefs across three waves and demonstrated that changes in beliefs appear to follow changes in behavior (Fotuhi et al., 2013; Sidhu et al., 2022). Smokers who successfully kicked the habit reported fewer rationalizations for continuing to light up thereafter. But there is a continued propensity to rationalize smoking among individuals who tried to quit but relapsed (Fotuhi et al., 2013). In cross-sectional studies, Oakes et al. (2004) proposed four types of self-exempt beliefs (“bulletproof,” “skeptic,” “jungle,” and “worth it”), all of which have negative associations with quitting progress (Oakes et al., 2004). Some beliefs are more strongly associated with the intention to cease than others (Huang et al., 2019). “Worth-it” beliefs, in particular, are strong independent predictors of smokers who do not intend to stop (Oakes et al., 2004). Skeptic beliefs (SB) are also revealed as the only variables significantly associated with the intention to quit (Guillaumier et al., 2016).

Previous studies have employed several methodologies, including the Health Belief Model (HBM), Knowledge, Attitudes, and Practices (KAP), and zero-inflated models, to identify the determinants of tobacco control. However, these studies have encountered some drawbacks, such as poor statistical significance, inadequate study scope, and incomplete identification of influencing factors (Ma et al., 2023). The protection motivation theory (PMT) is a robust theoretical framework that can effectively forecast the intentions of individuals to quit smoking (Yan et al., 2014; Xu and Chen, 2016; Lin and Chang, 2021; Lin et al., 2022). It was introduced by Rogers (1975), a prominent social psychologist, in the year 1975. It explains the process of behavioral change through threat appraisal (TA) and coping appraisal (CA) of cognitive regulation and offers a complete analysis of the internal mechanisms and processes involved in behavioral change from the perspective of influencing motivation. In recent years, the theory has been used in health education projects such as smoking cessation, long-term exercise, AIDS prevention, etc. (Maddux and Rogers, 1983; Milne et al., 2000).

The presence of self-exempt beliefs (despite its various appellations, the term “self-exempt beliefs” is consistently employed throughout this article) has been found to have detrimental consequences, both directly and indirectly, on individuals’ intentions to stop smoking (Zhang L. et al., 2023). PMT commonly employs TA and CA as cognitive mediators to impact external variables and intentions. Although earlier studies examined the impact of self-exempt beliefs and PMT constructs on the intention to quit smoking (IQS) separately, there was little focus on the relationship among self-exempt beliefs, PMT constructs, and the IQS. To help fill the evidence gap, we proposed that self-exempt beliefs could influence quit intention through the mediating roles of TA and CA. In this study, it was postulated that beliefs serve as enduring individual traits (Fotuhi et al., 2013), and it was not anticipated that beliefs would undergo modifications concurrent with changes in an individual’s behaviors. Therefore, the goal of this study was to examine how self-exempt beliefs affect smoking cessation intention and whether TA and CA played a mediating role in that process. The available evidence may have the potential to enhance the effectiveness of smoking cessation interventions for individuals who smoke.

## 2 Research model and hypotheses

**FIGURE 1:**
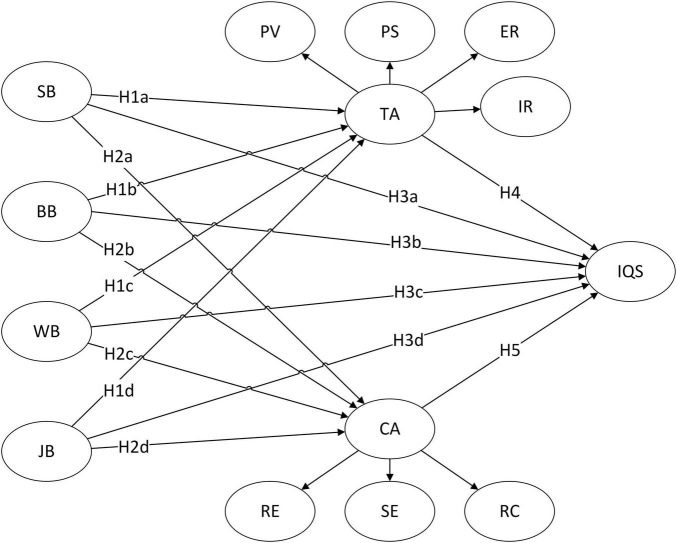
Conceptual framework. SB, skeptic beliefs; BB, bulletproof beliefs; WB, “worth it” beliefs; JB, jungle beliefs; TA, threat appraisal; CA, coping appraisal; IQS, intention to quit smoking.

To better understand the IQS, we integrated self-exempt beliefs and PMT to explore the effect of self-exempt beliefs on cessation intention and the mediating role of TA and CA. Figure 1 depicts the conceptual framework, which contains all the theoretical hypotheses of the present research. Based on the PMT framework, this study used four types of self-exempt beliefs as exogenous constructs of the model; TA and CA as cognitive mediation processes, and IQS as a dependent variable.

Self-exempt beliefs, which contribute to the development of an optimistic bias, are postulated to function as psychological mechanisms that mitigate perceptions of vulnerability or susceptibility to harm, a crucial driver of behavioral modification in theoretical frameworks like the PMT (Rogers et al., 1983; Borland et al., 2009). Hence, it is apparent that self-exempt beliefs have a detrimental influence on TA in the PMT. In addition, self-exempt beliefs can cause individuals to stop responding to negative behaviors, and it is reasonable to assume that these beliefs also have a negative effect on CA in PMT. According to the view of Oakes et al. (2004), self-exempt beliefs about smoking cessation have been classified into four categories, namely SB (doubting that tobacco is really as harmful as studies have shown), WB (ignoring the harms of tobacco and believing that smoking is worth these supposed risks), bulletproof beliefs (BB; believing that these harms or problems are not applicable to oneself), and jungle beliefs (JB; believing that the harms of smoking are normal and that the dangers are universal). The present study posited that there are four types of beliefs that are postulated to influence TA and CA, just as Kaplan et al. (2022) once hypothesized. The following hypotheses are developed in this study:

H1a: SB have a negative effect on TA;

H2a: SB have a negative effect on CA;

H1b: BB have a negative effect on TA;

H2b: BB have a negative effect on CA;

H1c: WB have a negative effect on TA;

H2c: WB have a negative effect on CA;

H1d: JB have a negative effect on TA;

H2d: JB have a negative effect on CA.

Self-exempt beliefs influence people’s decisions to engage in an action even when they are aware of the consequences (Yang et al., 2014). Specifically, it reduces the cognitive dissonance and anxiety experienced by smokers (Festinger, 1957), thus stopping them from quitting smoking (Oakes et al., 2004; xPeretti-Watel et al., 2007b). Previous studies have verified a negative correlation between smoking cessation tendencies and self-exempt beliefs (Guillaumier et al., 2016). Self-exempt beliefs are one of the predictors of success in quitting, and the stronger the self-exempt beliefs, the less likely the smoker is to quit (Oakes et al., 2004; Kasza et al., 2013). It is justifiable to assume that these beliefs have a negative effect on intentions to quit smoking. Based on the analysis, the following hypotheses are developed in this study:

H3a: SB have a negative effect on IQS;

H3b: BB have a negative effect on IQS;

H3c: WB have a negative effect on IQS;

H3d: JB have a negative effect on IQS.

According to PMT, the health protection intentions and behaviors of an individual are determined by two processes. One process that serves as an assessment of maladaptive behaviors is TA. It is based on a person’s perceptions of the health threat’s negative effects (perceived severity), their perceived susceptibility to its potential consequences (perceived vulnerability), and the advantages of engaging in maladaptive behavior (intrinsic and extrinsic rewards) (Lin et al., 2022). If perceived severity and vulnerability are high and rewards are low, one can anticipate stronger motivation for certain health behaviors (like the desire to cease smoking). Another process for achieving the protective aim is CA, which assesses an individual’s capacity to effectively manage and mitigate the potential hazard. The effectiveness of the preventative behavior in averting the threatened event, also known as response efficacy, is determined by an individual’s perceptions. Additionally, an individual’s confidence in carrying out preventative behavior, referred to as self-efficacy (Agaku et al., 2021), and the barriers associated with doing so, known as response costs, also play a significant role. It is anticipated that response costs will reduce coping evaluation, whereas response efficacy and self-efficacy will enhance coping evaluation (Lee et al., 2009). To sum up, TA encompasses perceived vulnerability, perceived severity, intrinsic rewards, and extrinsic rewards, while CA includes response efficacy, self-efficacy, and response costs.

Some literature did not directly incorporate TA and CA within the model. Instead, the model solely encompasses the variables of perceived vulnerability, perceived severity, intrinsic rewards, extrinsic rewards, response efficacy, self-efficacy, and response costs (Plotnikoff et al., 2010; Xiao et al., 2014; Wei et al., 2022). Subsequently, some academic works saw TA and CA as second-order constructs, leaving out variables like intrinsic rewards, extrinsic rewards, and response costs that were shown to have negative effects (Zhang et al., 2021; Zhang D. et al., 2023). Nevertheless, the current study incorporated intrinsic and extrinsic rewards as well as response costs, rendering it unsuitable for the establishment of second-order constructs. Hence, this study treated TA and CA as first-order constructs, and each of them was measured by a measurement variable whose value was calculated. The calculation formula elucidates the relationship among the internal variables of TA and CA. The value of the measurement variable of the TA construct was calculated as the difference between the mean of perceived vulnerability and perceived severity and the mean of internal and external rewards, whereas the value of the measurement variable of the CA construct was calculated as the difference between the mean of response efficacy and self-efficacy and response cost (Yan et al., 2014; Lin et al., 2022). Therefore, this means that there is no regression relationship between TA and its sub-variables, as well as between CA and its sub-variables.

Individuals’ intentions to perform protection behaviors are influenced by cognitive mediating processes known as TA and CA, which together make up the PMT (Rogers, 1975; Floyd et al., 2000). In previous studies, TA and CA were demonstrated as mediation factors, such as mediators between perceived destination support and social distancing intention (Zhang et al., 2021), between media trust and vaccination intention (Li and Sun, 2021), and so on. While the exogenous variables impacting TA and CA may vary, it is important to note that certain factors, such as knowledge, past experiences, and awareness, may serve as exogenous variables (Xiao et al., 2014). Self-exempt beliefs pertain to subjective cognitions that are grounded in objective knowledge and intimately intertwined with personal experiences. Hence, it is reasonable to assume that self-exempt beliefs have an impact on the IQS under the mediating effect of TA and CA. Based on the aforementioned analyses, the following hypotheses are developed in this study:

H4: TA has a positive effect on IQS;

H5: CA has a positive effect on IQS;

H6a: TA mediates the effect of SB on IQS;

H6b: TA mediates the effect of BB on IQS;

H6c: TA mediates the effect of WB on IQS;

H6d: TA mediates the effect of JB on IQS;

H7a: CA mediates the effect of SB on IQS.

H7b: CA mediates the effect of BB on IQS.

H7c: CA mediates the effect of WB on IQS.

H7d: CA mediates the effect of JB on IQS

## 3 Materials and methods

### 3.1 Measures

The content validity of the items was ensured by adapting and translating relevant pieces of existing literature into Chinese. It was discussed and amended several times by a few researchers. The instruments were then modified via a preliminary test. With some minor revisions, the model consists of 14 constructs for a total of 47 items, as shown in Supplementary Table 1.

#### 3.1.1 Self-exempt beliefs

The self-exempt beliefs comprise four subscales, such as SB (five items), BB (five items), WB (three items), and JB (four items), adapted from Oakes et al. (2004). One of the items of SB was measured by asking “Lots of doctors and nurses smoke, so it cannot be all that harmful.” One of the items of BB was measured by asking “Cancer mostly strikes people with negative attitudes.” One of the items of WB was measured by asking “I would rather live a shorter life and enjoy it than a longer one where I would be deprived of the pleasure of smoking.” One of the items of JB was measured by asking “Everything causes cancer these days.” All question items were measured using a seven-point Likert scale. The available response options spanned a scale of 1 (“extremely disagree”) to 7 (“extremely agree”).

#### 3.1.2 The components of the PMT

The components of PMT include perceived vulnerability, perceived severity, extrinsic rewards, intrinsic rewards, response efficacy, self-efficacy, response cost, and IQS. All other components of PMT comprise three items based on the scale created by MacDonell (2013) with the exception of intention to stop smoking, which has four items borrowed from Johnson et al.’s (2022) scale. Despite the fact that the scale developed by MacDonell (2013), was originally intended for Chinese vocational high school students, Lin et al. (2022) found that it was also applicable to Chinese adults.

One of the measures of perceived vulnerability is the question “I would become addicted if I smoked.” One of the measures of perceived severity is the question “The earlier a person starts smoking, the greater the harm.” One of the measures of extrinsic rewards is the question “Smokers look cool and fashionable.” One of the measures of intrinsic rewards is the question “Smoking makes people feel comfortable.” One of the measures of response efficacy is the question “If I quit smoking, I will live a longer and healthier life.” One of the measures of self-efficacy is the question “I am confident that I can quit smoking successfully.” One of the measures of response cost is the question “A person may be isolated if they quit smoking.” One of the measures of IQS is the question “In the next 3 months, how likely will you quit smoking completely?” Except for the response options of intention to quit, which range from 1 (“absolutely impossible”) to 5 (“very likely”), the response options of the other components of PMT range from 1 (“extremely disagree”) to 7 (“extremely agree”).

### 3.2 Participants

The current smoking prevalence in China above age 15 in 2018 was 26.6%, with that of males (50.5%) higher than that of females (2.1%) (Chinese Center for Disease Control and Prevention [CDC], 2020; Zhang et al., 2022). China has one of the world’s largest disparities in smoking rates between males and females, which is mostly due to cultural norms (Hitchman and Fong, 2011). In contrast to the prevailing norms in Western societies, Chinese society exhibits a greater degree of acceptance toward male smokers while simultaneously displaying a lack of tolerance toward female smokers. The factors associated with smoking and smoking cessation in women exhibit greater complexity compared to those observed in men. Besides, due to the extremely low smoking prevalence among women, investigating female smokers is challenging and therefore excluded. Consequently, the participants in this study were recruited based on the following criteria: (1) males aged above 18; (2) individuals who were current smokers (individuals who have engaged in persistent or cumulative smoking for a duration of at least six months throughout their lifespan and have also smoked within the past 30 days); (3) individuals who smoked cigarettes; and (4) individuals who provided consent to participate in the survey.

### 3.3 Procedure

Due to constraints in project funding, time allocation, and available resources, this study was unable to carry out extensive random sampling surveys on a large scale. The process of gathering data from various regions or employing investigators in order to obtain adequate and representative samples is both laborious and costly (Memon et al., 2020). Moreover, in situations where a sample frame is not accessible, the utilization of probabilistic sampling techniques may pose difficulties (Memon et al., 2017). Consequently, the selection of a non-probability sampling method, specifically snowball sampling, was deemed appropriate for the study. Initially, the panelists selected suitable male smokers they knew as the initial sample based on the sample recruitment criteria. These individuals were then provided with the Tencent questionnaire (a professional questionnaire platform on the Internet) link to complete the questionnaire. Online questionnaire surveys were utilized for data collection due to their convenience and cost-effectiveness. Additionally, this method facilitates efficient data input and guarantees the maintenance of data integrity throughout the entirety of the investigative procedure (Shiau and Luo, 2012). Subsequently, each member of the initial sample was requested to recommend 2–4 additional smokers who satisfied the specified criteria to participate in the study and fill out the questionnaire. Then, the new participants were also requested to recommend suitable smokers so as to expand the sample size like a snowball. Due to its cost-effectiveness, efficiency, and ability to rapidly engage a large pool of participants who satisfy the study criteria, the snowball sampling method is frequently employed in research situations where it is difficult to obtain a clear and complete sampling frame. Even though this non-probability sampling method could introduce bias, it can be reduced by increasing the size of the initial sample and making sure that a wide range of people are included in the snowball sample. The research team intentionally chose a set of 50 initial samples from diverse social circles, each with distinct demographic characteristics and originating from various geographical regions. The inclusion of a diverse range of individuals in the initial sample contributes to the diversity observed in the subsequent snowball sample, thereby augmenting the overall representativeness of the sample.

During the process of collecting data, we provided participants with comprehensive details regarding the study and its purpose and ensured that their privacy and data security were protected. As an acknowledgment of their engagement, participants had the opportunity to receive a virtual red envelope containing RMB 3 via a Tencent questionnaire. After completing the data collection portion of the research, participants received informational materials on smoking cessation resources and support. The investigation was conducted from 21 February 2023 to 26 February 2023. In the end, 513 questionnaires were collected. The Ethics Committee of Guizhou Medical University authorized the study’s ethical evaluation, with approval number 2022-297.

### 3.4 Sample size

In order to obtain the minimum sample size, this study utilized G*Power 3.1.9.7 software for power analysis, which is used in the PLS-SEM model. It can determine the minimum sample size by considering the part of the model with the most predictive factors (Roldán and Sánchez-Franco, 2012; Hair et al., 2016). Select “F tests,” “Linear multiple regression: fixed model, *R*^2^ deviation from zero,” and “A priority: compute required sample size – given α, Power, and effect size.” The next step requires setting the effect size to 0.15, α to 0.05, and power to 0.80, which is the optimal setting for studies in the social sciences and the business world (Hair et al., 2016). Subsequently, input the number 6 as the number of predictors, which denotes the upper limit of arrows that are directed toward a dependent variable within the model. G*Power estimates that a sample size of 98 is necessary to detect a medium effect size (Memon et al., 2020). Additionally, a popular method for estimating sample sizes in the behavioral and social sciences is the Krejcie and Morgan table (KMT) (Krejcie and Morgan, 1970). Since this table applies to any specific population, no calculations are necessary. For populations greater than one million, the KMT recommends 384 samples (Krejcie and Morgan, 1970). Nonetheless, the investigators received 513 responses, so the sample size for this study was adequate.

### 3.5 Data analysis

#### 3.5.1 Data cleaning and exclusion

The Tencent questionnaire platform was used to collect questionnaires because it is more convenient for snowball sampling techniques. After importing the data from 513 samples into SPSS 26.0 for the purpose of data cleaning, 488 samples were eventually kept. Participants who failed to meet the predetermined inclusion criteria were eliminated. The inclusion criteria for this study necessitate that participants meet specific prerequisites, including being adult males, current smokers, smoking cigarettes, and providing informed consent to participate in the survey. The recommended participants were chosen by the panelists and the participants from the previous round through a snowball sampling technique according to the criteria. But in order to enhance the study’s validity, the questionnaire includes relevant questions to facilitate the exclusion of samples that do not meet the requirements. Participants who were female, under 18 years old, used tobacco products other than cigarettes, or were non-current smokers were excluded from the study. Furthermore, participants who had aberrant responses, such as “extremely agree,” “extremely disagree,” or a certain value with all items regardless of the item’s content, were eliminated. These anonymized data are stored in Supplementary Dataset 1. The frequency of socio-demographic characteristics such as age, education, residential area, occupation, and chronic diseases was determined using descriptive statistics and SPSS 26.0.

#### 3.5.2 Exploratory factor analysis

Then, we used exploratory factor analysis (EFA) on 17 self-exempt beliefs to determine if they represented the four hypothesized distinct structures (BB, SB, WB, and JB). The data was processed with SPSS 26.0. Kaiser–Meyer–Olkin (KMO) tests were used to evaluate the representativeness of the sample. If the KMO value is more than 0.60, then there is enough data to conduct an EFA (Kline, 1994). To determine whether the data were fit for factor analysis, we used the KMO and Bartlett’s tests. The data are appropriate for EFA if the KMO value is more than 0.90 and the significance level for Bartlett’s test of sphericity is less than 0.05. Principal component analysis (PCA) and principal axis factoring (PAF) are widely utilized techniques for extracting factors in EFA. PCA is appropriate for factor analysis to explain the maximum variance of the original data with the fewest factors. PAF is suitable for factor analysis to identify the data structure (Sun and Zhou, 2005). To make the results of factor analysis easier to understand, factor rotation was used. Orthogonal rotation (like Varimax rotation) and oblique rotation (like Promax rotation) are frequent rotation techniques. Promax rotation was chosen as the factor rotation method due to its ability to facilitate easy interpretation of the factors while maintaining a simple structure among them. Importantly, this method permits inter-factor correlations, which is more realistic (Sun and Zhou, 2005). The criteria for evaluation in EFA depend on factor loadings and explained variance. Although the minimum acceptable threshold for factor loadings is in the range of 0.30–0.40, values greater than 0.50 are typically thought to be of practical significance (Hair et al., 2016). Items with a maximum load of less than 0.3 or a difference between two cross-loads of less than 0.1 were excluded from the analysis. Additionally, in order to guarantee the accurate measurement of each factor, the common factor with fewer than three items was eliminated (Zhou et al., 2017). In the social science field, if the cumulative variance contribution rate can reach 60% or more, it means that the common factor is reliable (Hair et al., 2016).

#### 3.5.3 Partial least squares analysis

The model was analyzed with the partial least squares structural equation modeling (PLS-SEM). Since partial least squares (PLS) can handle both continuous and sequential scales, analyze complicated latent variable models (Hair et al., 2012), accept small samples, and be used for non-probability sampling (Ringle et al., 2012; Hair et al., 2017), the PLS-SEM method is deemed to be more appropriate for the present study.

Partial least squares was used to examine both the external and internal models and mediation analysis in this research. The analysis was conducted using the two-step technique suggested by Anderson and Gerbing (1988), using the SmartPLS 3.0 software. Outer model analysis involves assessing the model’s reliability and validity, whereas inner model analysis involves calculating and evaluating the path coefficients and explanatory power of the structural model. The goals of the two phases are to ensure the validity of the interrelationships between the constructs by ensuring their reliability and validity. In terms of software operation, the PLS algorithm is frequently employed for estimating the path coefficients and *R*^2^, and it is frequently combined with bootstrapping to determine the significance (*P*-value and *t*-value) of the path coefficients. To carry out mediation analysis, we adhered to the methodology outlined by Zhao et al. (2010). If the mediating effect *a* × *b* is significant and the direct effect *c* is significant, then *a* × *b* × *c* represents a complementary partial mediation when it is positive and a competitive partial mediation when it is negative. If the mediating effect *a* × *b* is significant but the direct effect *c* is not, there is only indirect mediation or full mediation. If the mediating effect *a* × *b* is not significant and the direct effect *c* is significant, then there is direct-only non-mediation. If the mediating effect *a* × *b* is not significant and the direct effect *c* is significant, then there is no-effect non-mediation (Zhao et al., 2010).

## 4 Results

### 4.1 Descriptive statistics analysis

As indicated in Table 1, presented below, the male respondents in the study were distributed across various age groups. Specifically, 120 individuals (24.6%) fell within the 18–24 year old category, 222 individuals (45.5%) were aged between 25 and 30 years, 116 individuals (23.8%) were aged between 31 and 40 years, 12 individuals (2.5%) were aged between 41 and 50 years, and 18 individuals (3.6%) were above 50 years old. In terms of educational attainment, 45 respondents (9.2%) reported having completed junior high school or below, 125 respondents (25.6%) reported being in high school or secondary school, 303 respondents (62.1%) reported being undergraduates or enrolled in college, and 15 respondents (3.1%) reported being graduate students or holding advanced degrees. A total of 391 individuals, accounting for 80.1% of the population, resided in urban areas, while 97 individuals, representing 19.9% of the population, resided in rural areas, including villages and towns. Out of the total sample population, 78 individuals (16.0%) were identified as civil servants or staff members of public institutions. Additionally, 201 individuals (41.2%) were classified as workers or employees of various enterprises. A total of 77 individuals (15.8%) were self-employed, while 72 individuals (14.8%) were enrolled as students. Out of the total sample size, 89 individuals (18.2%) were found to have chronic diseases, while the remaining 399 individuals (81.8%) did not exhibit any chronic diseases. In terms of the number of cigarettes smoked per day, 230 individuals (47.1%) smoked 10 cigarettes or less per day, 197 individuals (40.4%) smoked 11–20 cigarettes per day, and a few individuals smoked 20–30 and 31 or more cigarettes per day.

**TABLE 1:** Demographic statistics from samples.

Item	Frequency	Percentage (%)
Age		
18–24	120	24.6
25–30	222	45.5
31–40	116	23.8
41–50	12	2.5
>50	18	3.6
Education		
Junior high school and below	45	9.2
High school/secondary school	125	25.6
Undergraduate/college	303	62.1
Graduate student and above	15	3.1
Residential area		
Cities	391	80.1
Villages and towns	97	19.9
Occupation		
Civil servants or staff of public institutions	78	16.0
Workers or enterprise staff	201	41.2
Farmer	38	7.8
Self-employed	77	15.8
Students	72	14.8
Others	22	4.5
Chronic diseases		
Yes	89	18.2
No	399	81.8
Number of cigarettes smoked per day		
10 or less	230	47.1
11–20	197	40.4
21–30	51	10.5
31 or more	10	2.0

### 4.2 Exploratory factor analysis

Due to the fact that the self-exempt beliefs scale is based on foreign research and is seldom used among Chinese smokers, we used EFA to determine its structural validity. The KMO test yielded a value of 0.963, indicating high sampling adequacy. Additionally, Bartlett’s test of sphericity resulted in a chi-square value of 5,554.138 with a *P*-value of 0.000, suggesting that the data is suitable for factor analysis (Sun and Zhou, 2005). The PAF method was chosen as the factor extraction method, which can be suitable for factor analysis to identify the data structure. During factor extraction, the desired number of factors was set to four. Promax rotation was chosen as the factor rotation method. The coefficients are displayed in descending order, and variables with loadings greater than 0.4 are retained for scale measurement item reduction through the pattern matrix. The results show that Factor 4 only contains JB3 (it is dangerous to walk across the street). Factor 4 is deleted due to its few variables and components. For the next round of factor extraction, the desired number of factors to be extracted is set to 3. Multiple explorations can be conducted during EFA. When the factor loading coefficient of an indicator variable is negative or less than 0.4, when the attribution of an indicator variable to a common factor contradicts the predetermined common factors, or when the factor loading of an indicator variable is very close to two common factors with a difference of less than 0.1, indicators must be eliminated.

After multiple rounds of EFA and indicator reduction, a total of six indicators (JB1, JB2, JB3, JB4, BB2, and BB4) were eliminated from the original 17 indicators, leaving only 11 indicators. This produced three common factors and acceptable outcomes. The variance explained by the factors before and after rotation is shown in Table 2, and the cumulative variance contribution rate reached 74.902% for the three extracted common factors, indicating that three common factors can explain most of the total variance. The pattern matrix is presented in Table 3, displaying the extracted three common factors: SB (consisting of 5 items), WB (consisting of 3 items), and BB (consisting of 3 items). The factor loadings of each indicator on the common factors are above 0.4, and the minimum acceptable factor loading is 0.30–0.40 (Hair et al., 2016), which means it has good representativeness.

**TABLE 2:** Explained variances of factors before and after rotation.

Factor	Initial eigenvalues			Sum of squared loadings			Sum of squared rotated loadings
	Total	Variance (%)	Cumulative (%)	Total	Variance (%)	Cumulative (%)	Total
1	6.596	59.968	59.968	6.266	56.964	56.964	5.787
2	0.899	8.170	68.138	0.464	4.217	61.181	5.275
3	0.744	6.765	74.902	0.331	3.006	64.187	4.706

**TABLE 3:** Factor loadings of exploratory factor analysis for the self-exempt beliefs scale (*n* = 488).

Items	Common factor	Factor loadings		
		1	2	3
SB1	Skeptic beliefs	0.892		
SB3		0.864		
SB5		0.795		
SB2		0.774		
SB4		0.545		
WB2	“Worth it” beliefs		0.787	
WB3			0.736	
WB1			0.731	
BB1	Bulletproof beliefs			0.667
BB3				0.661
BB5				0.455

### 4.3 Measurement model assessment

Partial least squares structural equation modeling requires a two-stage testing process, including measurement model assessment and structural model assessment (Anderson and Gerbing, 1988). The first stage is mainly for the outer model testing, such as the reliability, the convergent validity testing, and the discriminant validity testing of each construct (Jiang et al., 2023). Previous studies suggested that each indicator’s factor loadings must exceed 0.6; each factor’s reliability (CR, Cronbach’s α, and *rho-A*) must exceed 0.6; and each factor’s average variance extracted (AVE) must exceed 0.5 (Fornell and Larcker, 1981; Hair et al., 2016).

As shown in Table 4, except for the fact that there is only one indicator each for TA and CA, it can be seen that the standardized factor loadings of all the remaining items fall within the range of 0.706–0.917 and are statistically significant (*t* > 1.96); the values of CR of all the other constructs are between 0.833 and 0.943; the Cronbach’s α values range from 0.725 to 0.924; the *rho-A* values range from 0.795 to 0.938; and the AVE values fall within the range of 0.614 to 0.767. Hence, the study’s convergent validity exhibited positive outcomes (Chin, 1998).

**TABLE 4:** Reliability and convergent validity.

Construct	Items	Standardized factor loading	*T*-value	Composite reliability	AVE	Cronbach’s α	*rho-A*
SB	SB1	0.876	58.046	0.943	0.767	0.924	0.928
	SB2	0.847	43.905				
	SB3	0.892	79.297				
	SB4	0.869	66.801				
	SB5	0.892	92.946				
BB	BB1	0.738	12.648	0.833	0.628	0.725	0.938
	BB3	0.706	10.385				
	BB5	0.917	36.388				
WB	WB1	0.887	70.555	0.907	0.764	0.845	0.846
	WB2	0.882	59.372				
	WB3	0.853	45.453				
TA	TA1	1	0	1	1	1	1
CA	CA1	1	0	1	1	1	1
IQS	IQS1	0.797	30.513	0.864	0.614	0.791	0.795
	IQS2	0.801	33.593				
	IQS3	0.768	27.286				
	IQS4	0.766	28.075				

SB, skeptic beliefs; BB, bulletproof beliefs; WB, “worth it” beliefs; TA, threat appraisal; CA, coping appraisal; IQS, intention to quit smoking.

The existence of differences between constructs was evaluated by utilizing the discriminant validity test (Jiang et al., 2023). The Fornell-Larcker criterion matrix (Hu et al., 2022), as illustrated in Table 5, is a method commonly used to ascertain discriminant validity. The row or column values of each construct are found to be below the diagonal value, which means that the square roots of the AVE for each construct exhibit higher values compared to their correlation coefficients with the other constructs, indicating that the discriminant validity threshold has also been met (Fornell and Larcker, 1981; Hair et al., 2019). Additionally, according to Fornell and Larcker (1981), each scale item’s standardized factor loadings for its designated latent constructs are greater than their cross-loadings on any other constructs, indicating a reasonable degree of discriminant validity (Hair et al., 2016). Table 6 shows that all shaded standardized factor loadings are greater than cross loadings on any other construct, indicating good discriminant validity. Furthermore, Henseler et al. (2015) suggested the Heterotrait-Monotrait ratio (HTMT) as an indicator to assess discriminant validity. Values that fall below the thresholds of 0.85 or 0.9 are regarded as indicative of favorable discriminant validity. Table 7 shows a minimum value of 0.841, which falls below the threshold of 0.85, indicating strong discriminant validity.

**TABLE 5:** Fornell-Larcker criterion matrix.

	BB	CA	IQS	SB	TA	WB
BB	0.793					
CA	−0.35	1				
IQS	−0.11	0.303	0.783			
SB	0.725	−0.487	−0.218	0.876		
TA	−0.22	0.496	0.285	−0.362	1	
WB	0.689	−0.496	−0.288	0.746	−0.369	0.874

**TABLE 6:** Standardized factor loadings and cross loadings of the outer model.

	SB	BB	WB	CA	TA	IQS
SB1	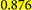	0.588	0.622	−0.44	−0.295	−0.219
SB2	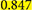	0.615	0.596	−0.352	−0.295	−0.134
SB3	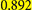	0.621	0.662	−0.441	−0.336	−0.212
SB4	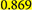	0.712	0.689	−0.437	−0.351	−0.174
SB5	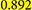	0.634	0.689	−0.45	−0.304	−0.208
BB1	0.492	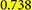	0.47	−0.217	−0.093	−0.042
BB3	0.442	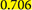	0.427	−0.187	−0.098	0.024
BB5	0.714	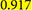	0.675	−0.367	−0.261	−0.162
WB1	0.696	0.603	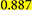	−0.454	−0.316	−0.265
WB2	0.66	0.636	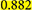	−0.434	−0.299	−0.261
WB3	0.598	0.566	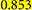	−0.412	−0.354	−0.228
CA1	−0.487	−0.35	−0.496	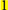	0.496	0.303
TA1	−0.362	−0.22	−0.369	0.496	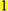	0.285
IQS1	−0.108	−0.01	−0.171	0.235	0.242	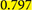
IQS2	−0.289	−0.189	−0.29	0.3	0.232	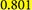
IQS3	−0.065	−0.045	−0.178	0.187	0.204	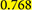
IQS4	−0.193	−0.083	−0.25	0.215	0.213	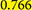

The shaded part represents the standardized factor loadings.

**TABLE 7:** Heterotrait-Monotrait ratio (HTMT).

	BB	CA	IQS	SB	TA	WB
BB						
CA	0.375					
IQS	0.147	0.336				
SB	0.836	0.504	0.242			
TA	0.22	0.496	0.319	0.376		
WB	0.833	0.54	0.347	0.841	0.402	

### 4.4 Structural model assessment

**FIGURE 2:**
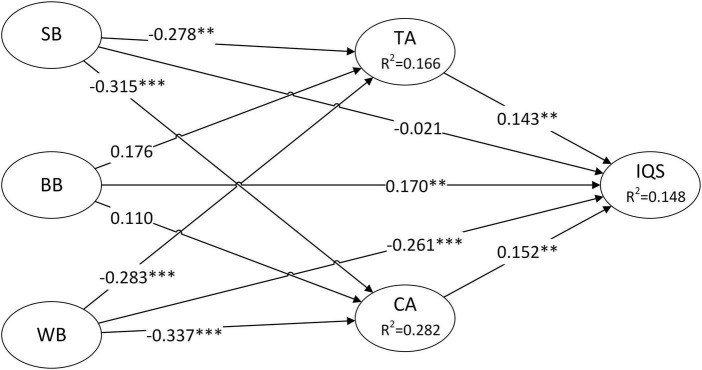
Inner model and path coefficient. SB, skeptic beliefs; BB, bulletproof beliefs; WB, “worth it” beliefs; TA, threat appraisal; CA, coping appraisal; IQS, intention to quit smoking. **P-value < 0.01; ***P-value < 0.001.

The subsequent stage of PLS testing is structural model assessment through a 10,000-time bootstrapping procedure. The standardized path coefficients along with their corresponding *T*-statistics, *P*-values, and hypotheses testing outcomes are presented in Table 8. In addition, Figure 2 visualizes relationships among the latent variables and their explanatory power (*R*^2^ values). Figure 2 and Table 8 display eight significant paths. Among all the self-exempting beliefs, it has been observed that SB exert a notable and adverse influence on both TA (H1a: β = −0.278) and CA (H2a: β = −0.315), but without significant influence on IQS; WB also demonstrate a negative and significant impact on TA (H1c: β = −0.283), CA (H2c: β = −0.337), and IQS (H3c: β = −0.261); whereas BB had no significant relationship with either TA or CA but a positive and significant impact on IQS (H3b: β = 0.170). Among all the PMT constructs, both TA (H4: β = 0.143) and CA (H5: β = 0.152) were positively and significantly related to IQS. Hence, with the exception of H3a, H1b, and H2b, all other hypothesis paths were endorsed.

**TABLE 8:** Summary of hypotheses testing results.

Hypothesis	Path	Standardized path coefficient	*T*-value	Supported
H1a	SB→TA	−0.278**	3.166	Yes
H2a	SB→CA	−0.315***	4.156	Yes
H3a	SB→IQS (c)	−0.021	0.275	No
H1b	BB→TA	0.176	1.873	No
H2b	BB→CA	0.110	1.601	No
H3b	BB→IQS (c)	0.170**	2.670	Yes
H1c	WB→TA	−0.283***	3.686	Yes
H2c	WB→CA	−0.337***	4.927	Yes
H3c	WB→IQS (c)	−0.261***	3.535	Yes
H4	TA→IQS	0.143**	3.552	Yes
H5	CA→IQS	0.152**	4.494	Yes

**P-value < 0.01; ***P-value < 0.001.

The *R*^2^ value, which indicates the proportion of the dependent variable that can be explained, was used to estimate the model’s explanatory power (Hair et al., 2016; Chen and Lin, 2019). As shown in Figure 2, the *R*^2^ values for TA and CA were determined to be 0.166 and 0.282, respectively, indicating the combined influence of SB, BB, and WB accounts for 16.6% of the variability in TA and 28.2% of the variability in CA. Moreover, the *R*^2^ value of IQS is 0.148, indicating TA and CA together explain 14.8% of the variability in IQS. According to Chin (1998), an endogenous latent variable exhibiting an *R*^2^ value of approximately 0.33 signifies a moderate level of explanatory power, while an *R*^2^ value of around 0.19 suggests a weak level of explanatory power. In another study, the *R*^2^ value of quitting intention was 0.16 (Lin and Chang, 2021), which closely aligns with the level observed in the present study. Hence, the model exhibited a modest yet normal degree of explanatory capacity.

### 4.5 Mediation analysis

In order to conduct mediation analysis, we followed the procedures proposed by Zhao et al. (2010) for mediation analysis. As shown in Table 9, the indirect effect of SB on IQS through TA (*a* × *b*) is significant (β = −0.04, *t* = 2.085). Then, as shown in Table 8, the direct effect of SB on IQS (c) is not significant (H3a: β = −0.021, *t* = 0.275). Therefore, indirect-only mediation was confirmed, and H6a was supported. Likewise, the mediating impact of H7a yields identical outcomes. Furthermore, the indirect effect of BB on IQS through TA (*a* × *b*) is not significant (β = −0.025, *t* = 1.495). But the direct effect of BB on IQS (c) is significant (H3b: β = 0.170, *t* = 2.670). Thus, direct-only non-mediation was confirmed, and H6b was rejected. Similarly, H7b was rejected too. In addition, the indirect effect of WB on IQS through TA (*a* × *b*) is significant (β = −0.041, *t* = 2.167). The direct effect of WB on IQS (c) is also significant (β = −0.261, *t* = 3.535). The indirect effect and indirect effect operate in the same direction (*a* × *b* × *c* is positive). Consequently, complementary mediation was confirmed, and H6c was supported. H7c similarly exhibits the identical mediating effect.

**TABLE 9:** Mediating effect.

Hypothesis	Indirect effects (a × b)	Standardized path	*T*-value	*P*-value	Mediating effect type	Supported
H6a	SB→TA→IQS	−0.04	2.085	0.037	Indirect-only	Yes
H7a	SB→CA→IQS	−0.048	2.4	0.016	Indirect-only	Yes
H6b	BB→TA→IQS	0.025	1.495	0.135	Direct-only	No
H7b	BB→CA→IQS	0.017	1.36	0.174	Direct-only	No
H6c	WB→TA→IQS	−0.041	2.167	0.03	Complementary	Yes
H7c	WB→CA→IQS	−0.051	2.519	0.012	Complementary	Yes

## 5 Discussion

### 5.1 Main findings

The present research investigated the influence of smokers’ self-exempt beliefs on TA, CA, and IQS within the framework of PMT. Through EFA, the three-factor structure of self-exempt beliefs, namely SB, BB, and WB, was determined, while JB were excluded. TA and CA, as two main cognitive processes, acted as full mediations between SB and the intention to quit, as complementary partial mediations between WB and the intention, and as non-mediation between BB and the intention.

This study utilized the self-exempt scale developed by Oakes et al. (2004) and the PMT scale developed by MacDonell (2013). Despite the fact that Oakes et al. (2004) discovered a four-factor structure for the self-exempting beliefs scale, this structure was slightly inappropriate for the study’s data. This research identified a three-factor structure for self-exempting beliefs by EFA, including SB, BB, and WB. This result aligns with the findings of Kortenkamp et al. (2021). In the factor rotation process, the indicators of “everything causes cancer these days,” “the government would ban tobacco sales if smoking was so bad for you,” and “smoking is no more risky than lots of other things that people do” are easily rotated by the same common factor as indicators of WB. But the indicator “it is dangerous to walk across the street” always rotated independently under a common factor. This suggests that the JB exhibit no substantial difference from the other three beliefs or that their significance is less than the other three. It is different from Oakes et al.’s (2004) research, which demonstrated that JB were most closely related to the enactment of the decision to quit.

Skeptic beliefs had a notable adverse effect on both TA and CA, with a slightly greater impact on CA than on TA, but did not directly affect smoking cessation intention. These findings suggested that TA and CA acted as full mediations in the association between SB and the IQS. Smokers with strong SB may not have an explicit intention to stop smoking, but they are more likely to come up with a variety of justifications for why they think smoking is not as harmful, such as the fact that many healthcare professionals are also smokers and that many smokers live long lives, which leads them to believe smoking poses less threat. Most importantly, the more skepticism smokers hold, the less confident they are in their ability to quit successfully. Both pathways reduce the likelihood of quitting smoking. This indicated a good agreement with Guillaumier et al.’s (2016) study that SB were significant predictors of smoking cessation.

It was also indicated that WB had a significant negative influence on TA and CA, even slightly more than SB. Notably, unlike SB, WB have a direct and substantial negative effect on smoking cessation intentions. Thus, TA and CA both acted as complementary partial mediations between WB and the IQS. These beliefs of smokers reflect their values of pursuing happiness. WB induce paranoia in smokers that smoking is the important path to happiness, so they directly undermine the intention to quit and impede their cessation behavior. In addition, individuals who engage in smoking exhibit a preference for immediate gratification derived from the act of smoking rather than contemplating the potential adverse health consequences that may arise in the long run (Pei and Yang, 2022), resulting in a low TA. Furthermore, WB can be understood as an individual’s evaluation of costs and benefits (Oakes et al., 2004), fostering the perception that “smoking is worth it, while quitting is not worth it,” consequently heightening the response costs associated with quitting smoking, diminishing the response efficacy, and thus reducing the CA. Both direct and indirect effects strengthened the negative impact on intention, which made WB the most powerful predictor.

However, different from the initial assumptions, the study discovered that BB had a positive and non-significant effect on both TA and CA but a significantly positive effect on IQS, which means the more powerful their BB, the more likely it is that they will develop a desire to quit smoking. Smokers with strong BB did not believe that catastrophe would befall them since they believed that they smoked less, had a positive attitude, ate healthy food, and exercised regularly to resist the negative consequences of smoking. But these beliefs did not significantly improve threat or CAs; a possible explanation for this might be that they are less robust in establishing self-exempt beliefs than expected, consistent with Oakes et al.’s (2004) research.

Consistent with previous studies, both threat and response appraisals demonstrated significant positive predictive value for IQS (Lin and Chang, 2021). Moreover, it is also verified that the impact of CA is slightly greater than that of TA (Zhang et al., 2021; Lin et al., 2022), indicating that smokers quit not primarily due to their fear of diseases caused by smoking but rather due to their conviction in the advantages of cessation and feeling confident in their capacity to successfully quit. Regarding the PMT variables, all the results are in accordance with the viewpoint of the PMT.

### 5.2 Implications

The findings of the present investigation hold significant theoretical and practical ramifications. In theory, this is the first study to explore the relationship between self-exempt beliefs and threat and CA within the framework of PMT. This study explored how self-exempt beliefs influence IQS and the mediating role of TA and CA, which had not been studied earlier. It expanded both the PMT and the self-exempt belief theory of smoking. Furthermore, the PLS-SEM method proved particularly applicable in dealing with this model, yet it was rarely used in previous related research.

In practice, our research has implications for activities or interventions aimed at preventing tobacco use. Current promotion messages for quitting smoking in China emphasize the detrimental effects of smoking on both the smoker’s own and others’ health (Pei and Yang, 2022), but smoking rates have not decreased significantly. The current study revealed the pervasive, deep-rooted perceptions owned by smokers that contribute to their resistance to quitting. Many smokers hold self-exempt beliefs that justify their hazardous behaviors. These doubts and beliefs are inaccurate and become huge obstacles to quitting smoking. Therefore, relevant authorities should make efforts to undermine or “prevent” these self-exempt beliefs, particularly WB and SB, which may be an effective strategy for health communication interventions for smoking cessation. Specifically, it is possible to rectify the misleading WB and SB through targeted media interventions so as to encourage quitting.

### 5.3 Limitations

There were several constraints associated with this study. First, the survey used snowball sampling through social networks to recruit participants, which allowed for the rapid collection of sample data but suffered from selection bias and had limited representativeness of the population as a whole. Second, due to the small number of individuals aged 41 and older who frequently smoke for longer durations and may have a lower propensity to quit, the results may differ from those of samples aged 40 and younger, thereby limiting the generalizability of the findings. Moreover, excluding women from the study may weaken its explanatory power. The study’s scope was restricted to male smokers; therefore, it is important to exercise caution when generalizing the results to female smokers in China.

## 6 Conclusion

The primary goal of the current research was to investigate the impact of self-exempt beliefs on the intention to cease smoking and the mediating role of TA and CA in it. Different from previous studies, it established a model containing self-exempt beliefs, PMT constructs, and the IQS. This study has provided a deeper insight into self-exempt beliefs. It indicated that two types of self-exempt beliefs, namely SB and WB, had a negative and significant effect on both TA and CA. This research broadened the meaning and application of the self-exempt belief theory and PMT. It also enriched the current literature about quit intentions among smokers. Our findings suggested that efforts to undermine or “prevent” these self-exempt beliefs may be an effective strategy for health communication interventions for smoking cessation. This research offers significant evidence supporting effective policies and programs to boost quit rates among Chinese smokers.

## Data availability statement

The original contributions presented in this study are included in this article/Supplementary material, further inquiries can be directed to the corresponding author.

## Ethics statement

The studies involving humans were approved by the Ethics Committee of Guizhou Medical University. The studies were conducted in accordance with the local legislation and institutional requirements. The participants provided their written informed consent to participate in this study.

## Author contributions

DZ: Conceptualization, Formal analysis, Funding acquisition, Methodology, Writing – original draft. RL: Supervision, Validation, Writing – review and editing. XL: Formal analysis, Resources, Validation, Visualization, Writing – original draft. YY: Data curation, Investigation, Writing – review and editing. GZ: Conceptualization, Funding acquisition, Project administration, Writing – review and editing.
