# Supplementary material for: How do self-exempt beliefs affect intentions to quit smoking? An exploration of the mediating role of threat appraisal and coping appraisal
Source: Front Psychol. 2023 Nov 30;14:1260561. doi: 10.3389/fpsyg.2023.1260561 (PMC10720447; doi:10.3389/fpsyg.2023.1260561)
Supplement: Supplementary file 1 [file Data_Sheet_1.docx]

Supplementary Material

## Supplementary Tables

Table S1. Measurement items of constructs

| **Construct** | **Source** | **Indicator code** | **Measurement item** |
| --- | --- | --- | --- |
| Skeptic beliefs | Oakes, W et al. (2004) [1] | SB1 | Lots of doctors and nurses smoke, so it cannot be all that harmful. |
|  |  | SB2 | The medical evidence that smoking is harmful is exaggerated. |
|  |  | SB3 | Smoking cannot be all that bad for you because many people who smoke live long lives. |
|  |  | SB4 | Smoking cannot be all that bad because some top athletes smoke and still perform well. |
|  |  | SB5 | More lung cancer is caused by such things as air pollution, petrol, and diesel fumes than smoking. |
| Bulletproof beliefs | Oakes, W et al. (2004) | BB1 | Cancer mostly strikes people with negative attitudes. |
|  |  | BB2 | They will have found cures for cancer and all the other problems smoking causes before I am likely to get any of them. |
|  |  | BB3 | You can overcome the harms of smoking by doing things like eating healthy food and exercising regularly. |
|  |  | BB4 | I think I must have the sort of good health or genes that mean I can smoke without causing any harm. |
|  |  | BB5 | I think I would have to smoke a lot more than I do to put my health at risk. |
| ‘‘Worth it’’ beliefs | Adapted from Oakes, W et al. (2004) | WB1 | I would rather live a shorter life and enjoy it than a longer one where I would be deprived of the pleasure of smoking. |
|  |  | WB2 | You have got to die of something, so why not enjoy yourself and smoke? |
|  |  | WB3 | Life is short, and smoking brings more pleasure than harm. |
| Jungle beliefs | Oakes, W et al. (2004) | JB1 | Everything causes cancer these days. |
|  |  | JB2 | The government would ban tobacco sales if smoking was so bad for you. |
|  |  | JB3 | It is dangerous to walk across the street. |
|  |  | JB4 | Smoking is no more risky than lots of other things that people do. |
| Perceived vulnerability | MacDonell, K. (2013) [2] | PV1 | I would become addicted if I smoked. |
|  |  | PV2 | I would get sick if I smoked. |
|  |  | PV3 | If I smoked, I may have died earlier. |
| Perceived severity | MacDonell, K. (2013) | PS1 | The earlier a person starts smoking, the greater the harm. |
|  |  | PS2 | More smokers get sick than nonsmokers. |
|  |  | PS3 | Smokers die earlier than nonsmokers. |
| Extrinsic rewards | MacDonell, K. (2013) | ER1 | Smokers look cool and fashionable. |
|  |  | ER2 | Smoking is good for social networking. |
|  |  | ER3 | The life of a smoker is happier than that of a nonsmoker. |
| Intrinsic rewards | MacDonell, K. (2013) | IR1 | Smoking makes people feel comfortable. |
|  |  | IR2 | Smoking helps people concentrate. |
|  |  | IR3 | Smoking enhances brainwork. |
| Response efficacy | MacDonell, K. (2013) | RE1 | If I quit smoking, I will live a longer and healthier life. |
|  |  | RE2 | People will be less likely to get diseases if they do not smoke. |
|  |  | RE3 | Quitting smoking is good for disease recovery. |
| Self-efficacy | MacDonell, K. (2013) | SE1 | I am confident that I can quit smoking successfully. |
|  |  | SE2 | I have the ability to stop smoking. |
|  |  | SE3 | I think stopping smoking is easy for me. |
| Response cost | MacDonell, K. (2013) | RC1 | A person may be isolated if they quit smoking. |
|  |  | RC2 | Refusing a cigarette offer is very impolite. |
|  |  | RC3 | One will miss the enjoyment if he or she quits smoking. |
| IQS | Johnson, A.C. et al (2022) [5] | IQS1 | In the next 3 months, how likely will you quit smoking completely? |
|  |  | IQS2 | In the next 3 months, how likely will you reduce the number of cigarettes you smoke? |
|  |  | IQS3 | In the next 3 months, how likely will you refrain from smoking in the near future? |
|  |  | IQS4 | In the next 3 months, how likely will you talk to a friend, family member, spouse, or partner about quitting smoking? |

1. Oakes, W. ‘“Bulletproof Skeptics in Life’s Jungle”’: Which Self-Exempting Beliefs about Smoking Most Predict Lack of Progression towards Quitting? *Preventive Medicine* **2004**, *39*, 776–782, doi:10/bzm6f7.

2. MacDonell, K. A Protection Motivation Theory-Based Scale for Tobacco Research among Chinese Youth. *J Addict Res Ther* **2013**, *04*, doi:10/gjd953.

3. Jankowski, M.; Pinkas, J.; Zgliczyński, W.S.; Kaleta, D.; Wierzba, W.; Gujski, M.; Rees, V.W. Voluntary Smoke-Free Home Rules and Exposure to Secondhand Smoke in Poland: A National Cross-Sectional Survey. *Int J Environ Res Public Health* **2020**, *17*, 7502, doi:10/grmcft.

4. Thompson, T.; Evbuoma-Fike, E.I.; Garg, R.; McQueen, A.; Caburnay, C.; Kreuter, M.W. Examining Psychosocial Correlates of a Home Smoking Ban Among Low-Income Smokers: Analysis of Social Support, Unmet Social Needs, Perceived Stress, and Depressive Symptoms. *J Community Health* **2022**, *47*, 959–965, doi:10/grxrck.

5. Johnson, A.C.; Turner, M.M.; Simmens, S.J.; Evans, W.D.; Strasser, A.A.; Mays, D. Mediational Effects on Motivation to Quit Smoking After Exposure to a Cigarette Pictorial Warning Label Among Young Adults. *Ann Behav Med* **2022**, *56*, 737–748, doi:10/grsr6f.
